# It's all in whom you know (as well as what you know). Using network analysis and health professionals own knowledge for recruitment

**DOI:** 10.1186/1745-6215-14-S1-P86

**Published:** 2013-11-29

**Authors:** Charlene McShane, Olinda Santin, Helen McAneney, Michael Donnelly, Jackie Quinn, Oonagh Sheehy, Liam Murray, Lesley Anderson

**Affiliations:** 1 Cancer Epidemiology Health Services Research Group, Centre for Public Health, Queen’s University, Belfast, UK; 2 School of Nursing and Midwifery, Queen’s University Belfast, Belfast, UK; 3 MRC Methodology Hub, Centre for Public Health, Queen’s University Belfast, Belfast, UK; 4 Institute of Child Care Research, Queen’s University Belfast, Belfast, UK; 5 Belfast City Hospital, Belfast Health and Social Care Trust, Belfast, UK

## 

Often the decision of whom to interview within a study is crucial to its ultimate success or failure, but when the study is to assess a very specific subset of health professionals, this decision may be even more vital.

One avenue that has been proposed within the AiMS Study, is to make use of the knowledge that the group of interest inherently possesses, the names of others working with the specific patient group. Using the tools of network analysis, the frequency and linkage of the names given will be analysed to assess who are the key or central persons whom the study should recruit for interview. Techniques will include degree, betweenness and eigenvector centrality, as while as core-periphery structure analysis of the network graph. The benefits of this approach are that rather than interviewing a large or random sample of health professionals, the core individuals are approached, reducing the number of interviews to be conducted, and in theory, conducting interviews with those having greater knowledge of the specific patient group. The protocol, ethical considerations and success of such a stratum for recruitment will be discussed.

